# Bioactive Cembrane-Based Diterpenoids from the Soft Coral *Sinularia triangular*

**DOI:** 10.3390/md9060944

**Published:** 2011-05-27

**Authors:** Jui-Hsin Su, Zhi-Hong Wen

**Affiliations:** 1 National Museum of Marine Biology & Aquarium, Pingtung 944, Taiwan; 2 Graduate Institute of Marine Biotechnology, National Dong Hwa University, Pingtung 944, Taiwan; 3 Department of Marine Biotechnology and Resources, National Sun Yat-sen University, Kaohsiung 804, Taiwan; E-Mail: wzh@mail.nsysu.edu.tw

**Keywords:** soft coral, *Sinularia triangular*, cytotoxicity, anti-inflammatory

## Abstract

Chemical examination of the Taiwanese soft coral *Sinularia triangular* led to the isolation of five cembrane-based diterpenoids **1**–**5**, including two new metabolites, triangulenes A (**1**) and B (**2**). The structures of the new metabolites were determined on the basis of extensive spectroscopic analysis, particularly mass spectroscopy and 2D NMR (^1^H–^1^H COSY, HMQC, HMBC, and NOESY) spectroscopy. Metabolites **3** and **5** exhibited moderate cytotoxicity to human tumor cell lines CCRF-CEM and DLD-1. Furthermore, **3**–**5** displayed significant *in vitro* anti-inflammatory activity in lipopolysaccharide-stimulated RAW264.7 macrophage cells by inhibiting the expression of the iNOS protein. Metabolites **4** and **5** also effectively reduced the expression of the COX-2 protein in the macrophages.

## 1. Introduction

**Figure 1:**
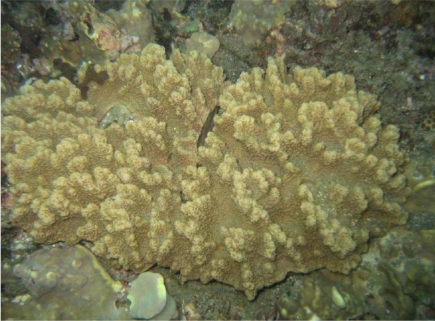
Soft coral *Sinularia triangular*.

Our previous chemical examination of soft corals of the genus *Sinularia* led to the isolation and identification of various oxygenated cembrane-type metabolites [1–5]. Some of these metabolites exhibit anti-inflammatory activity [2–4] and/or cytotoxicity to the growth of some cancer cell lines [1]. Our ongoing research to discover bioactive metabolites from the soft coral *Sinularia triangular* (Tixier-Durivault, 1970; family Alcyoniidae) (Figure 1) led to the isolation of two new cembrane-based diterpenoids, triangulenes A (**1**) and B (**2**), along with three known metabolites, sinularin (**3**) [6], dihydrosinularin (**4**) [6], and (−)14-deoxycrassin (**5**) [7]. The structures of **1** and **2** were established by extensive spectroscopic analysis, including 2D NMR spectroscopy. The cytotoxicity of **1**–**5** to the human tumor cell lines CCRF-CEM (T-cell acute lymphoblastic leukemia) and DLD-1 (colon adenocarcinoma) was studied, and the ability of **1**–**5** to inhibit the expression of the pro-inflammatory iNOS (inducible nitric oxide synthase) and COX-2 (cyclooxygenase-2) proteins in lipopolysaccharide (LPS)–stimulated RAW264.7 macrophage cells was also evaluated.

## 2. Results and Discussion

**Figure 2:**
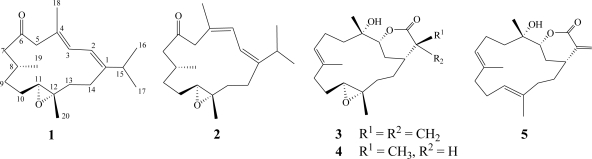
Structures of **1**–**5**.

Frozen samples of *S. triangular* were extracted with EtOAc. The dry EtOAc extracts were fractionated by silica gel gravity column chromatography, and the eluted fractions were further purified by HPLC to yield cembranoids **1**–**5** (Figure 2).

**Figure 3:**
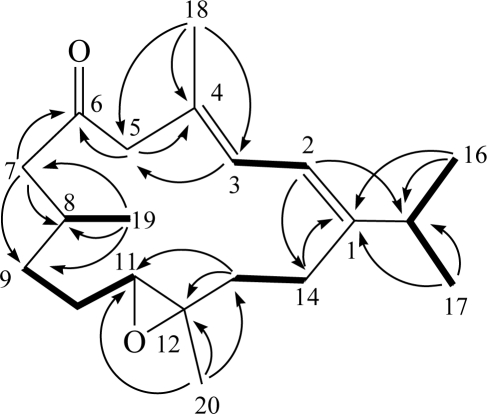
Key ^1^H–^1^H COSY and HMBC correlations of **1**.

**Figure 4:**
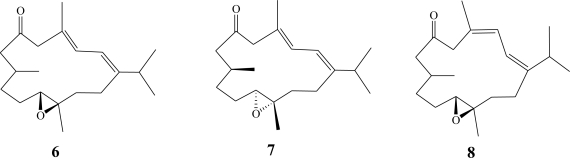
Structures of **6**–**8**.

The HRESIMS spectrum of triangulene A (**1**) contained a molecular ion peak consistent with the molecular formula C_20_H_32_O_2_, indicating the molecule has five double-bond equivalent. A UV absorption maxima at 240 nm (log*ɛ* = 4.0) was attributed to double bond conjugation. The IR spectrum of **1** revealed the presence of a carbonyl functionality (ν_max_ = 1703 cm^−1^). The ^13^C NMR data of **1** showed the presence of 20 carbons (Table 1): five methyls, six sp^3^ methylenes, three sp^3^ methines (including an oxygenated carbon at δ 62.7), two sp^2^ methines, and four quaternary carbons (including an oxygenated carbon at δ 61.5, two olefinic carbons with resonances at δ 148.3 and δ 129.3, and a keto-carbonyl at δ 209.8). The ^1^H NMR data revealed the presence of two olefinic methine protons as doublets at δ 6.15 and δ 6.06. A proton signal at δ 2.71 (1H, dd, *J* = 8.4, 4.0 Hz) that correlated with a carbon signal at δ 62.7 in the HMQC spectrum of **1** was attributed to the proton of a trisubstituted epoxide. The gross planar structure of **1** was determined by detailed analysis of its 1D and 2D NMR spectra. From the ^1^H–^1^H COSY correlations (Figure 3), it was possible to establish five partial structures of consecutive proton spin systems extending from H-2 to H-3; H-8 to H_3_-19; H_2_-9 to H-11; H_2_-13 to H_2_-14; and H-15 to H_3_-16 and H_3_-17. The following key HMBC correlations permitted connection of the carbon skeleton: H-2 to C-1, C-14, and C-15; H-3 to C-5; H-5 to C-4 and C-6 (carbonyl carbon); H-7 to C-6, C-8, and C-9; H-13 to C-11 and C-12; H_3_-16 and H_3_-17 to C-1 and C-15; H_3_-18 to C-3, C-4, and C-5; H_3_-19 to C-7, C-8, and C-9; and H_3_-20 to C-11, C-12, and C-13. Thus, **1** was found to possess a tetrasubstituted diene at C-1/C-2 and C-3/C-4, a ketone group at C-6, and a trisubstituted epoxide at C-11/C-12. The above results indicate that **1** possessed the same molecular framework as known cembranoids **6** and **7** (Figure 4), which were isolated previously from octocorals *Eunicea tourniforti* [8] and *Eunicea* sp. [9], respectively.

**Figure 5:**
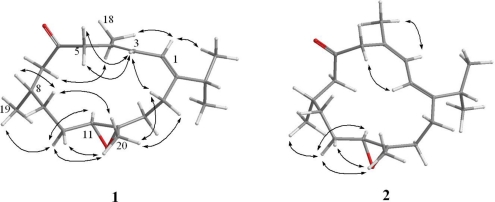
Selective NOESY correlations of **1** and **2**.

The relative configuration of **1** was determined from NOE correlations observed in the NOESY spectrum (Figure 5). The NOE correlations between H-2 and methyl protons H_3_-16 and H_3_-18 and between H-3 and H_2_-5 indicated *E* configurations for the double bonds at C-1/C-2 and C-3/C-4. In addition, one proton of C-10 methylene (δ 1.92) was found to exhibit correlations with H-11 (δ 2.71, dd, *J* = 8.4, 4.0 Hz) and H_3_-19 (δ 0.93, d, *J* = 6.8 Hz), indicating that these protons were situated on the same face; they were assigned as α protons, as C-20 methyl was β-oriented at C-12, which were verified by the absence of correlation between H-11 and H_3_-20. Furthermore, H_3_-20 correlated with protons of C-10 (δ 1.92 and 1.14) and C-14 (δ 2.37 and 2.28) methylenes, respectively. Consideration of molecular models found that H_3_-20 was reasonably close to H_2_-10 and H_2_-14 when H_3_-20 was β-oriented. Based on the above findings, the structure of **1**, including its relative configuration was established, and the chiral centers for **1** were assigned as 8S*, 11S*, and 12S*. Furthermore, the chemical shifts of **1** were shifted downfield at C-7 (Δ*δ_C_* +1.7 ppm) and C-8 (Δ*δ_C_* +2.5 ppm) and upfield at C-19 (Δ*δ_C_* −0.8 ppm) relative to the corresponding chemical shifts of **7**. On the basis of the above findings, we determined the relative structure of **1**, which was determined to be the C-8 epimer of **7**.

Triangulene B (**2**) had the same molecular formula (C_20_H_32_O_2_) as **1**, as indicated by HRESIMS and NMR spectra (Table 1). Comparison of the ^1^H and ^13^C NMR data of **2** with those of **1** revealed that the two compounds possessed similar structures. The trisubstituted double bonds at C-1/C-2 and C-3/C-4 of **2** had *Z* geometries, as indicated by NOE interactions (Figure 5) between H-3 (δ 6.29) and H_3_-18 (δ 1.93) and between H-2 (δ 6.19) and H-5 (δ 3.90). After determining the structure of **2**, we discovered that its planar structure has been obtained previously as diterpenoid **8** from the octocoral *Eunicea* sp. [8]. Furthermore, we found that the NMR data for **2** were similar to those of **8**, except that C-7 and C-8 of **2** were shifted markedly downfield (Δδ_C_ +3.9 ppm and Δδ_C_ +3.6 ppm, respectively) relative to the corresponding carbons of **8**. Further analysis of other NOE interactions revealed that **1** and **2** possessed the same relative configurations at C-8, C-11, and C-12. Thus, the structure of **2** was established unambiguously.

**Figure 6:**
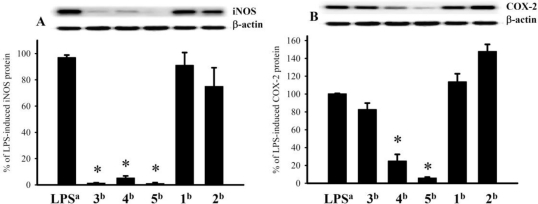
Immunoblot analysis of the effects of **1**–**5** (10 μM) on the expression of the iNOS and COX-2 proteins of RAW264.7 macrophage cells: (**A**) Immunoblots of iNOS and β-actin and (**B**) immunoblots of COX-2 and β-actin. The relative intensity for the cells stimulated with LPS alone was set at 100%. Band intensities were quantified by densitometry and are indicated as percentages relative to the intensities for the LPS-stimulated cells. Western blotting with β-actin was performed to verify that equivalent amounts of protein were loaded in each lane. Values represent mean ± SEM (*n* = 6). *Significantly different from the values for cells stimulated with LPS alone (*P < 0.05). *^a^*Stimulated with LPS alone; *^b^*stimulated with LPS in the presence of **1**–**5**.

Study of the cytotoxicity of diterpenoids **1**–**5** to human tumor cell lines CCRF-CEM and DLD-1 showed that **3** and **5** moderately inhibited the growth of the tested cell lines (the ED_50_ values were 26.0 and 37.1 μM for **3** and 29.8 and 32.2 μM for **5** for CCRF-CEM and DLD-1, respectively). The *in vitro* anti-inflammatory effects of **1**–**5** were also tested. The inhibition of LPS-stimulated upregulation of the pro-inflammatory proteins iNOS and COX-2 in RAW264.7 macrophage cells was measured by immunoblot analysis. At a concentration of 10 μm, **3**–**5** reduced the levels of the iNOS protein to 1.2 ± 0.3%, 5.1 ± 1.6%, and 0.9 ± 0.7%, respectively, of the levels in control cells stimulated with LPS alone (set at 100%). At the same concentration, **4** and **5** markedly reduced the levels of COX-2 to 24.9 ± 7.4% and 5.9 ± 1.0%, respectively, relative to controls (Figure 6).

## 3. Experimental Section

### 3.1. General Experimental Procedures

Melting points were measured on Fargo apparatus and are uncorrected. Optical rotation values were measured with a Jasco P-1010 digital polarimeter. Ultraviolet spectra were recorded on a Jasco V-650 spectrophotometer. IR spectra were obtained with a Varian Digilab FTS 1000 FT-IR spectrophotometer. NMR spectra were recorded with a Varian Mercury Plus 400 FT-NMR, at 400 MHz for ^1^H NMR and 100 MHz for ^13^C NMR, in CDCl_3_. ESIMS and HRESIMS data were recorded with a Bruker APEX II mass spectrometer. Silica gel 60 (230–400 mesh; Merck, Darmstadt, Germany) was used for column chromatography. Gravity column chromatography was performed on silica gel (230–400 mesh; Merck). TLC was carried out on precoated Kieselgel 60 F254 (0.2 mm; Merck), and spots were visualized by spraying with 10% H_2_SO_4_ solution followed by heating. HPLC was performed on a system comprising a Hitachi L-7100 pump, a Hitachi photodiode array detector L-7455, and a Rheodyne 7725 injection port. A semi-preparative reverse-phase column (Hibar 250 × 10 mm, LiChrospher 100 RP-18e, 5 μm, Merck) and a preparative normal-phase column (Hibar 250 × 21 mm, Si-60 column, 7 μm, Merck) were used for HPLC.

### 3.2. Animal Material

The marine soft coral *S. triangular* (specimen No. 200807-15) was collected by scuba divers at a depth of around 10 m off the coast of Taitung County, Taiwan, in July 2008, and the samples were frozen immediately after collection. A voucher sample was deposited at the Department of Marine Biotechnology and Resources, National Sun Yat-sen University, Taiwan.

### 3.3. Extraction and Separation

The frozen bodies of *S. triangular* (1.2 kg, wet weight) were minced and exhaustively extracted with EtOAc (1 L × 5). The combined EtOAc extracts (15.5 g) were subjected to silica gel column chromatography with elution by EtOAc in *n*-hexane (0–100%, stepwise) followed by 100% acetone; and the fractions were pooled on the basis of TLC analysis to yield 17 fractions. Fraction 8 (265 mg), which eluted with *n*-hexane–EtOAc (10:1), was subjected to silica gel column chromatography with gradient elution (*n*-hexane–acetone, 12:1 to 6:1) to afford five subfractions (A1–A5). Subfraction A2 (20 mg) was subjected to reverse-phase HPLC with MeOH–H_2_O (5:1) elution to afford **1** (2.5 mg) and **2** (2.0 mg). Subfraction A3 (90 mg) was subjected to normal-phase HPLC using *n*-hexane–acetone (10:1) to afford **5** (50.3 mg). Fraction 11 (160 mg), which eluted with *n*-hexane–EtOAc (5:1), was subjected to silica gel column chromatography with gradient elution (*n*-hexane–acetone, 8:1 to 5:1) to yield six subfractions (B1–B6). Subfraction B3 was subjected to normal-phase HPLC with *n*-hexane–acetone (7:1) elution to afford **3** (20.5 mg) and **4** (10.8 mg).

Triangulene A (**1**): colorless oil; [α]^25^_D_ +70.8 (*c* 0.5, CHCl_3_); IR (neat) *v*_max_ 2961, 2928, 1703, 1456, 1385, and 1261 cm^−1^; UV (MeOH) λ_max_ 240 (log ɛ = 4.0); ^13^C and ^1^H NMR data, see Table 1; ESIMS *m/z* 327 [M + Na]^+^; HRESIMS *m/z* 327.2302 [M + Na]^+^ (calcd for C_20_H_32_O_2_Na, 327.2300).

Triangulene B (**2**): colorless oil; [α]^25^_D_ +50.6 (*c* 0.5, CHCl_3_); IR (neat) *v*_max_ 2959, 2928, 1709, 1460, and 1385 cm^−1^; UV (MeOH) λ_max_ 239 (log ɛ = 3.8); ^13^C and ^1^H NMR data, see Table 1; ESIMS *m/z* 327 [M + Na]^+^; HRESIMS *m/z* 327.2301 [M + Na]^+^ (calcd for C_20_H_32_O_2_Na, 327.2300).

Sinularin (**3**): white powder; mp 151–153 °C; [α]^25^_D_ −120 (*c* 0.5, CHCl_3_); ESIMS *m/z* 357 [M + Na]^+^ [6].

Dihydrosinularin (**4**): white powder; mp 116–118 °C; [α]^25^_D_ –42 (*c* 0.3, CHCl_3_); ESIMS *m/z* 359 [M + Na]^+^ [6].

(−)14-Deoxycrassin (**5**): colorless oil; [α]^25^_D_ −15 (*c* 1.0, CHCl_3_); ESIMS *m/z* 341 [M + Na]^+^ [7].

### 3.4. Cytotoxicity Testing

The cytotoxicity of **1**–**5** to CCRF-CEM and DLD-1 tumor cells was evaluated by means of the tetrazolium-based colorimetric assay [10,11]. As a positive control, we employed doxorubicin, which exhibited cytotoxicity to CCRF-CEM and DLD-1 cells with ED_50_ values of 0.57 and 0.25 μm, respectively.

### 3.5. *In Vitro* Anti-Inflammatory Assay

A macrophage (RAW264.7) cell line was purchased from ATCC. We measured the *in vitro* anti-inflammatory activities of **1**–**5** by examining the inhibition of LPS-simulated upregulation of the iNOS (inducible nitric oxide synthetase) and COX-2 (cyclooxygenase-2) proteins in macrophages using western blotting analysis [12,13].

## Figures and Tables

**Table 1:** ^1^H and ^13^C NMR data for **1** and **2**.

	**1**		**2**	
	^1^H*^a^*	^13^C*^b^*	^1^H*^a^*	^13^C*^b^*
1		148.3 (C)		146.7 (C)
2	6.06 d (10.8)	118.2 (CH)	6.19 d (10.8)	118.7 (CH)
3	6.15 d (10.8)	125.7 (CH)	6.29 d (10.8)	124.4 (CH)
4		129.3 (C)		130.5 (C)
5	3.20 d (13.6); 3.06 d (13.6)	54.7 (CH_2_)	3.90 d (13.6); 2.70 d (13.6)	48.9 (CH_2_)
6		209.8 (C)		208.0 (C)
7	2.54 dd (13.2, 8.4); 2.17 m	51.2 (CH_2_)	2.52 m; 2.12 m	52.4 (CH_2_)
8	2.03 m	31.2 (CH)	1.83 m	32.0 (CH)
9	1.48 m; 1.18 m	33.1 (CH_2_)	1.31 m; 1.14 m	33.3 (CH_2_)
10	1.92 m; 1.14 m	26.4 (CH_2_)	1.88 m; 1.09 m	26.4 (CH_2_)
11	2.71 dd (8.4, 4.0)	62.7 (CH)	2.49 m	64.4 (CH)
12		61.5 (C)		60.6 (C)
13	2.15 m; 1.32 m	36.7 (CH_2_)	2.12 m; 1.36 m	36.7 (CH_2_)
14	2.37 m; 2.28 m	25.9 (CH_2_)	2.73 m; 2.12 m	25.7 (CH_2_)
15	2.35 m	32.2 (CH)	2.35 m	30.8 (CH)
16	1.06 d (7.2)	22.6 (CH_3_)	1.18 d (6.8)	20.6 (CH_3_)
17	1.08 d (7.6)	22.1 (CH_3_)	1.02 d (6.8)	23.4 (CH_3_)
18	1.89 s	18.2 (CH_3_)	1.93 s	25.1 (CH_3_)
19	0.93 d (6.8)	19.5 (CH_3_)	0.85 d (6.4)	19.2 (CH_3_)
20	1.20 s	17.6 (CH_3_)	1.23 s	16.3 (CH_3_)

a Spectra were recorded at 400 MHz in CDCl_3_; *J* values (Hz) are given in parentheses;

b spectra were recorded at 100 MHz in CDCl_3_; attached protons were deduced by DEPT experiments.
